# Effect of Reproductive Stage-Waterlogging on the Growth and Yield of Upland Cotton (*Gossypium hirsutum*)

**DOI:** 10.3390/plants12071548

**Published:** 2023-04-03

**Authors:** Uzzal Somaddar, Shamim Mia, Md. Ibrahim Khalil, Uttam Kumer Sarker, Md. Romij Uddin, Md. Salahuddin Kaysar, Apurbo Kumar Chaki, Arif Hasan Khan Robin, Abeer Hashem, Elsayed Fathi Abd_Allah, Chien Van Ha, Aarti Gupta, Jong-In Park, Lam-Son Phan Tran, Gopal Saha

**Affiliations:** 1 Department of Agronomy, Patuakhali Science and Technology University, Dumki, Patuakhali 8602, Bangladesh; 2 Department of Agronomy, Bangladesh Agricultural University, Mymensingh 2202, Bangladesh; 3 On Farm Research Division, Bangladesh Agricultural Research Institute, Gazipur 1701, Bangladesh; 4 School of Agriculture and Food Sciences, The University of Queensland, St. Lucia, QLD 4072, Australia; 5 Department of Genetics and Plant Breeding, Bangladesh Agricultural University, Mymensingh 2202, Bangladesh; 6 Botany and Microbiology Department, College of Science, King Saud University, P.O. Box. 2460, Riyadh 11451, Saudi Arabia; habeer@ksu.edu.sa (A.H.); 7 Plant Production Department, College of Food and Agricultural Sciences, King Saud University, P.O. Box. 2460, Riyadh 11451, Saudi Arabia; eabdallah@ksu.edu.sa (E.F.A.); 8 Department of Plant and Soil Science, Institute of Genomics for Crop Abiotic Stress Tolerance, Texas Tech University, Lubbock, TX 79409, USA; 9 Department of Horticulture, Sunchon National University, Suncheon 57922, Republic of Korea

**Keywords:** cotton, ginning out turn, hypoxia, MDA, waterlogging, yield

## Abstract

The reproductive stage of cotton (*Gossypium* sp.) is highly sensitive to waterlogging. The identification of potential elite upland cotton (*Gossypium hirsutum*) cultivar(s) having higher waterlogging tolerance is crucial to expanding cotton cultivation in the low-lying areas. The present study was designed to investigate the effect of waterlogging on the reproductive development of four elite upland cotton cultivars, namely, Rupali-1, CB-12, CB-13, and DM-3, against four waterlogging durations (e.g., 0, 3, 6, and 9-day). Waterlogging stress significantly impacted morpho-physiological, biochemical, and yield attributes of cotton. Two cotton cultivars, e.g., CB-12 and Rupali-1, showed the lowest reduction in plant height (6 and 9%, respectively) and boll weight (8 and 5%, respectively) at the highest waterlogging duration of 9 days. Physiological and biochemical data revealed that higher leaf chlorophyll, proline, and relative water contents, and lower malondialdehyde contents, particularly in CB-12 and Rupali-1, were positively correlated with yield. Notably, CB-12 and Rupali-1 had higher seed cotton weight (90.34 and 83.10 g, respectively), lint weight (40.12 and 39.32 g, respectively), and seed weight (49.47 and 43.78 g, respectively) per plant than CB-13 and DM-3 in response to the highest duration of waterlogging of 9 days. Moreover, extensive multivariate analyses like Spearman correlation and the principle component analysis revealed that CB-12 and Rupali-1 had greater coefficients in yield and physiological attributes at 9-day waterlogging, whereas CB-13 and DM-3 were sensitive cultivars in response to the same levels of waterlogging. Thus, CB-12 and Rupali-1 might be well adapted to the low-lying waterlogging-prone areas for high and sustained yield.

## 1. Introduction

Globally, the occurrence of flooding or waterlogging (WL) has been increasingly frequent and unpredictable in recent years because of climate change [1]. Water is highly essential for the growth, development, and performance of plants. However, excess water in the rooting zone of plants from flooding or seasonal heavy rainfalls negatively affects their growth and development [2,3]. The low oxygen (O_2_) condition (known as hypoxia) under WL is considered as one of the major factors that affects plant performance [4,5]. Specifically, the limited exchange of gases (e.g., carbon dioxide (CO_2_) and O_2_) between soil rooting zones and atmosphere dramatically limits a number of physiological processes of plants, including water uptake, photosynthesis, and respiration [6]. Moreover, O_2_ deficiency under WL initiates various deleterious events, such as the inhibition of metabolic activities in plants, which causes the accumulation of by-products of fermentation in roots (e.g., ethanol and acetaldehyde), acid loads in cells [7], and various toxic compounds in soils (e.g., phenolic acid, hydrogen sulfide, volatile fatty acids, nitric oxide, methane, and CO_2_) [8]. It has been shown that WL changes the cation exchange capacity (CEC) of soil particles and valency of nutrients (more reduced forms), making them unavailable for plant uptake or even toxic for plants [9]. The nutrient deficiency caused by WL ultimately interrupts numerous biological processes, such as reducing root growth and shoot growth, stomatal closure, and causing chlorosis and necrosis leading to premature leaf senescence, inhibiting photosynthesis and respiration, and increasing susceptibility to diseases [10,11,12].

Cotton (*Gossypium* sp.) is an important cash crop, making significant contribution to fiber, feed, and oil productions [13]. The yearly economic value of cotton is nearly US $500 billion, providing income for about 100-million families in approximately 150 countries [14]. Among different cultivated species, the upland cotton (*G. hirsutum*) is the prime contributor of fiber (35%) for the textile industries worldwide [15]. Cotton is considered to be the second most important cash crop in Bangladesh after jute (*Corchorus capsularis*) [16]. Yearly, there is a huge demand of cotton fiber (8.6 million bales) for the textile industries in Bangladesh [17]. Bangladesh fulfills 95% of its cotton demand by import from India, USA, Australia, Brazil, Uzbekistan, and different African countries [17,18]. Currently, only about 0.5 million hectares of land are suitable for cotton cultivation in Bangladesh [19]. The low-lying south coastal regions have higher potential to expand cotton cultivation. However, there are two main challenges of cotton cultivation in the coastal areas, namely, (i) frequent tidal flooding of the low-lying cotton fields, and (ii) heavy rainfall during monsoon that coincides with the yield-determining reproductive growth stages of cotton [16]. Both these conditions create WL that causes significant yield losses or total failure of cotton cultivation [20].

Cotton is poorly adapted to WL [21,22,23]. Evidence suggests that the sensitivity of cotton plants to WL is mainly because of two reasons: (i) being unable to develop functional aerenchyma either lysigenously or naturally [12,24], and (ii) the generation of very low levels of alcohol dehydrogenases, which are associated with the detoxification of products of anaerobic metabolism [25]. WL causes significant reductions in root growth, restricts nutrient uptake [12,26,27], reduces leaf area, and inhibits photosynthesis [12,23], which together leads to reduced dry matter production in cotton [23,28].

The duration and depth of WL, as well as the growth stages of plants when they are exposed to WL, can considerably affect the growth, development, and performance of cotton plants [1,23,29,30]. Several studies showed that WL at squaring and flowering stages remarkably reduces the fiber yield by suppressing the development of cotton, where leaf growth, followed by plant height and stem diameter, is subjected to the largest inhibitory effect of WL [23,31,32]. WL negatively affects physiological attributes like stomatal conductance, leaf water potentials, and photosynthetic rates of cotton leaves [23,33,34,35]. A wealth of studies reported that chlorophyll content in the cotton leaves decreases with the increase in WL duration, resulting in a significant difference in the dry matter accumulation in the cotton bolls [29,30,36,37]. Many researchers reported that significantly higher percentages of reductions in the numbers of sympodial branches, flowers, bolls, as well as weight of boll and total yield were observed with the increase in WL duration in cotton [23,29,38]. Furthermore, some reports have highlighted that WL reduces cotton fiber quality by compromising the fiber length, strength and uniformity, lint percentage, and seed index [23,29,39].

Cotton, being an indeterminate plant species, is possibly better able to adapt to WL conditions [1,30,39]. Studies revealed that cotton plants can quickly perceive and gradually adapt to WL through morphological, biochemical, physiological, and molecular mechanisms to counteract the WL-induced loss of cotton productivity [1,28,29,30,34,39]. Therefore, on the above knowledge, the current study was undertaken to systematically assess the WL tolerance of four popular Bangladeshi elite upland cotton cultivars at their reproductive growth stages, with a view to identify suitable cultivar(s) adaptable to WL conditions to promote cotton cultivation in the low-lying WL-prone areas.

## 2. Results

### 2.1. Effect of WL on the Phenotypes and Growth Attributes of Cotton

**Figure 1:**
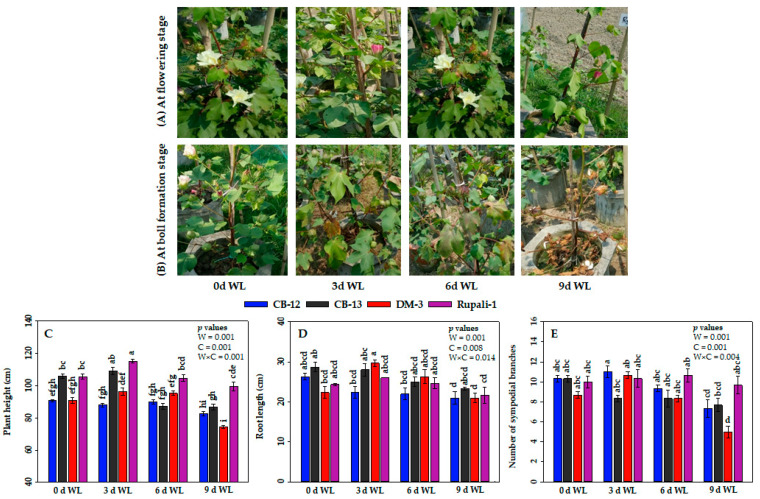
Phenotypic differences of cotton plants under different waterlogging (WL) conditions (**A**,**B**). Effect of WL on (**C**) plant height, (**D**) root length, and (**E**) number of sympodial branches. Values are means ± standard errors. Bars connected with different letters have significant differences. *p* values are presented at the top right corner of the graphs. W = WL duration, C = cotton cultivar and W × C = interaction of WL duration and cotton cultivar. d; day(s).

WL had a greater influence on the phenotypes and growth attributes, except root dry weight, of cotton plants (Figure 1A–E). Among the four cotton cultivars, Rupali-1 showed the highest plant height (115.3 cm) at 3 days (d) of WL, which was statistically similar with the plant height of CB-13 (109.3 cm). DM-3 incurred the highest reduction of 18% in plant height at 9 d of WL compared with the control (0-d WL) condition. CB-12 showed stability in plant height considering all WL durations (Figure 1C). In case of root length, the highest root length (29.7 cm) was noted in DM-3 at 3 d of WL (Figure 1D). The lowest root length (21 cm) was also observed in DM-3 at 9 d of WL exposure. Another three cultivars showed statistically similar root lengths in spite of the progression of WL durations. The number of sympodial branches of DM-3 showed the greatest extent of reduction (42%) at 9 d of WL as compared with control (Figure 1E).

**Figure 2:**
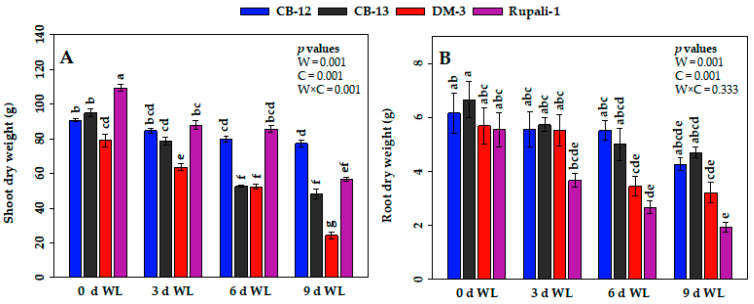
Effect of waterlogging (WL) on (**A**) shoot dry weight and (**B**) root dry weight. Values are means ± standard errors. Bars connected with different letters have significant differences. *p* values are presented at the top right corner of the graphs. W = WL duration, C = cotton cultivar, and W × C = interaction of WL duration and cotton cultivar. d; day(s).

Shoot and root dry weights were affected significantly in response to WL (Figure 2A,B). The highest shoot dry weight (109.3 g) was recorded in Rupali-1 at 0-d control (Figure 2B). The lowest shoot dry weight was observed in DM-3 (24.3 g) at 9 d of WL. Interestingly, shoot dry weight in Rupali-1 after 9 d of WL significantly decreased by 48% (Figure 2A). In case of root growth, all the cotton cultivars had statistically similar amounts of root dry weight up to 3-d WL, except for the Rupali-1, which showed reduced root dry weight by 34% compared with the respective value at 0 d (Figure 2B). As long as the duration of WL increased, the root dry weight of Rupali-1 continued to decrease, showing the highest reduction of 65% at 9 d of WL (Figure 2B), whereas CB-12 and CB-13 showed statistically similar root dry weights even at 9 d of WL, as compared with their respective value at 0-d control.

### 2.2. Effect of WL on the Physiological and Biochemical Parameters of Cotton Cultivars

#### 2.2.1. Proline Contents

**Figure 3:**
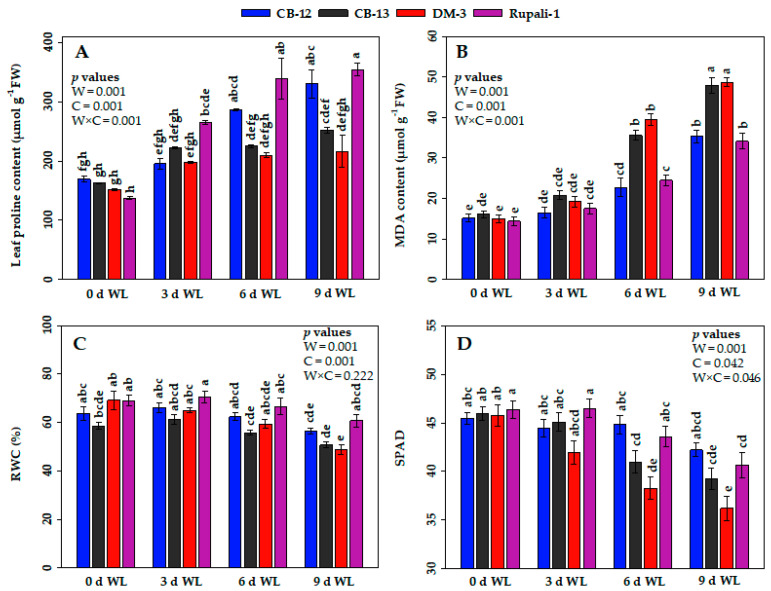
Effect of waterlogging (WL) on (**A**) leaf proline content, (**B**) malondialdehyde (MDA) content, (**C**) relative water content (RWC), and (**D**) SPAD values. Values are means ± standard error. Bars connected with different letters have significant differences. *p* values are presented at the top right corner of the graphs. W = WL duration, C = cotton cultivar, and W × C = interaction of WL duration and cotton cultivar. d, day(s); FW, fresh weight.

Cotton genotypes exposed to WL during reproductive developmental stages showed an increasing pattern of leaf proline contents with the increase of WL duration (Figure 3A). Briefly, at the 0-d WL, leaf proline contents in four elite cotton cultivars varied from 137.11 to 169.27 µmol g^−1^ fresh weight (FW), which were noted to be statistically similar and the lowest contents across all three WL regimes (3-d, 6-d and 9-d WL) (Figure 3A). Afterward, a significant increment of leaf proline contents in Rupali-1 and CB-12 was observed with the advance of the WL stress. At 3 d of WL, Rupali-1 had the highest leaf proline content (264.24 µmol g^−1^ FW), whereas the remaining three cotton cultivars showed statistically similar proline levels. From 6 d to 9 d of WL, Rupali-1 and CB-12 gradually upregulated their leaf proline levels, where CB-13 and DM-3 showed statistically non-significant increments of leaf proline contents compared with their respective value at 0 d. Considering all the WL regimes, the greatest proline content was observed in Rupali-1 (354.26 µmol g^−1^ FW) after 9 d of WL, followed by CB-12 (330.02 µmol g^−1^ FW), CB-13 (251.63 µmol g^−1^ FW), and DM-3 (215.87 µmol g^−1^ FW) (Figure 3A). Notably, Rupali-1 and CB-12 showed the greatest extent of increase in proline content by 158 and 95%, respectively, after 9 d of WL compared with their respective value at 0 d (Figure 3A).

#### 2.2.2. Malondialdehyde (MDA) Contents

Cotton cultivars exposed to 0 to 3 d of WL exhibited no significant differences in MDA content in cotton leaves (Figure 3B). However, at 6 d of WL stress, MDA contents in CB-13 and DM-3 remarkably increased by 20 and 24%, respectively, whereas those in Rupali-1 and CB-12 showed steadiness as compared with their respective value at 0 d. Similarly, at 9 d of WL stress, the highest and most statistically similar MDA contents were recorded from CB-13 (47.86 µmol g^−1^ FW) and DM-3 (48.74 µmol g^−1^ FW), whereas the lowest amounts of MDA contents observed from Rupali-1 (34.10 µmol g^−1^ FW) and CB-12 (35.28 µmol g^−1^ FW). Notably, we found that at the highest level of WL stress (9 d), Rupali-1 and CB-12 exhibited the lowest increment of MDA content by 20%, whereas CB-13 and DM-3 showed the greatest extent of MDA content by 32 and 34%, respectively, compared with their respective values at 0-d control (Figure 3B).

#### 2.2.3. Relative Water Contents (RWCs)

All the cotton cultivars showed statistically similar leaf RWCs from 0 d to 3 d WL (Figure 3C). Interestingly, at 3 d of WL, Rupali-1 had the highest RWC (71%), which was statistically on par with those of CB-12, CB-13, and DM-3 at 0 d. Afterwards, from 6 d to 9 d of WL, all cotton cultivars showed gradually declining trend in RWCs. Notably, at the highest level of WL stress of 9 d, Rupali-1 had the highest (61%) and DM-3 had the lowest leaf RWC (49%). Considering percent reductions in RWCs, at 9 d of WL, the highest reduction was observed in DM-3 (by 30%), followed by Rupali-1 (by 12%), CB-12 (by 11%), and CB-13 (by 13%) compared with their respective value at 0 d (Figure 3C).

#### 2.2.4. SPAD Reading Values

In general, the SPAD reading values in all the cotton genotypes were statistically similar at 0 d and 3 d of WL (Figure 3D). Afterwards, with the increase of the duration of WL, CB-12 and Rupali-1 showed remarkable stability in SPAD reading values till the 9 d of WL, whereas DM-3 showed a gradually decreasing trend of SPAD reading values by 8–21%, at 3 d, 6 d, and 9 d of WL. In addition, after having high and stable SPAD reading values at 0 d and 3 d of WL, CB-13 showed a gradual reduction in SPAD reading values at 6 d and 9 d of WL (Figure 3D).

### 2.3. Effect of WL on the Yield Attributes of Cotton Cultivars

**Figure 4:**
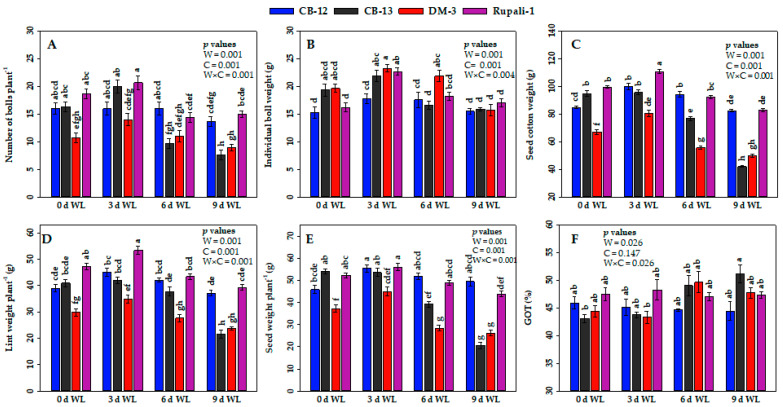
Effect of waterlogging (WL) on (**A**) number of bolls plant^−1^; (**B**) individual boll weight; (**C**) seed cotton weight; (**D**) lint weight plant^−1^; (**E**) seed weight plant^−1^; and (**F**) ginning out turn (GOT). Values are means ± standard error. Bars connected with different letters have significant differences. *p* values are presented at the top right corner of the graphs. W = WL duration, C = cotton cultivar and W × C = interaction of WL duration and cotton cultivar, d; day(s).

Various levels of WL significantly affected different yield characteristics of cotton, such as boll numbers, individual boll weight, seed cotton weight, lint weight, and seed weight (Figure 4). The dynamics of change in the boll numbers in response to WL followed similar patterns in all four cotton cultivars (Figure 4A). Generally, boll numbers in all four elite cotton cultivars decreased gradually when exposed to 6 d and 9 d WL. The highest number of bolls per plant (21) was obtained from Rupali-1 at 3 d of WL, which was statistically on par with those of Rupali-1, CB-12 and CB-13 at 0 d, and C-12 and CB-13 at 3 d of WL. Intriguingly, CB-12 had almost similar numbers of cotton bolls even at 6 d of WL as compared with those at 0 and 3 d of WL. At 9 d of WL, the reductions in boll numbers of CB-12, CB-13, DM-3, and Rupali-1 were recorded as 15, 55, 16, and 20%, respectively, as compared with their respective control at 0 d (Figure 4A). Furthermore, individual boll weight increased by 16, 13, 18, and 40% in CB-12, CB-13, DM-3, and Rupali-1, respectively, as compared with their respective control at 0 d (Figure 4B). Afterwards, at 6 d of WL, individual boll weight of CB-13 significantly decreased by 14%, whereas those of CB-12, DM-3, and Rupali-1 showed stability as compared with their respective control at 0 d. However, at 9 d of WL exposure, DM-3 and CB-13 displayed remarkably reduced boll weights by 20 and 18%, respectively, whereas CB-12 and Rupali-1 still maintained consistency in boll weight when compared with their respective control at 0 d, as observed throughout all the WL treatments (Figure 4B).

When looking into the variability in seed cotton yield, Rupali-1 showed the highest seed cotton weight per plant (110.71 g), even after being exposed to 3 d of WL (Figure 4C). Onward, at 6 d and 9 d of WL, Rupali-1 showed reductions in seed cotton weight by 7 and 16%, respectively, as compared with their respective control at 0 d. Moreover, CB-12 showed increased seed cotton weight by 18 and 11% respectively, after 3 d and 6 d of WL and only a slight reduction at 9 d of WL compared with their respective control at 0 d. After having stability in seed cotton weight up to 3 d of WL, CB-13 and DM-3 showed drastically reduced seed cotton weight with the progression of WL duration. Notably, the highest reduction (56%) was observed in CB-13 after 9 d of WL (Figure 4C).

Lint weights per plant of all four cotton cultivars were found to be the highest at 3 d WL, which was also consistent with most of the other estimated parameters (Figure 4D). Rupali-1 showed the highest lint weight (53.41 g) per plant at 3 d of WL over control. Then, the lint weights in Rupali-1 decreased gradually by 8 and 17%, respectively, at 6 d and 9 d of WL. Besides, a remarkably stable lint weights were observed from CB-12 throughout the WL duration, and CB-12 and Rupali-1 showed statistically similar lint weight values at 6 d and 9 d of WL. The lint weight per plant of CB-13 reduced drastically by 47% at 9 d of WL compared with their respective control at 0 d (Figure 4D). Furthermore, as like as in the case of lint weight, CB-12 had stable and statistically higher seed weight per plant (51.8 and 49.5 g at 6 d and 9 d of WL, respectively), which was followed by Rupali-1. At 9 d of WL, DM-3 and CB-13 showed the greatest extent of reduction in seed weight per plant by 30 and 62%, respectively, when compared with their respective 0-d control (Figure 4E). In the case of ginning out turn (GOT), statistically similar results were obtained for CB-12, DM-3, and Rupali-1 throughout the WL duration (Figure 4F). Our data collectively revealed that WL variably impacted all the growth and yield attributes of four cotton cultivars except GOT.

### 2.4. Correlation Analysis among the Growth and Yield Traits of Four Cotton Cultivars

**Figure 5:**
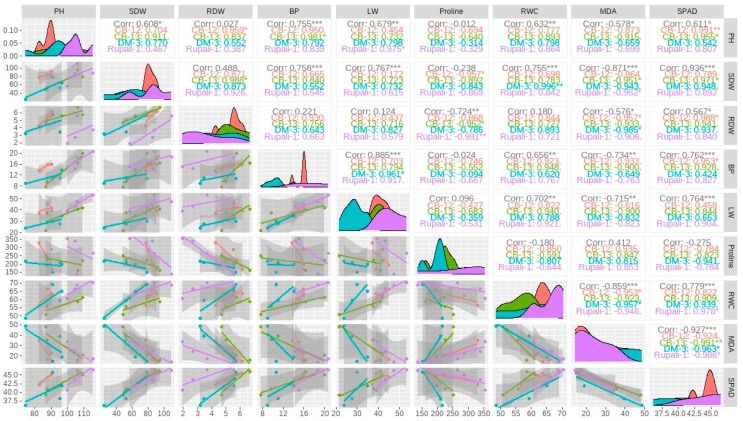
Spearman correlation matrix among the growth and yield traits of four investigated cotton cultivars. The upper triangle represents the type of relationships (significant or non-significant) and the lower part indicates the regression relationship with confidence level among the cotton cultivars. *, ** and *** indicate significant correlations at *p* < 0.05, *p* < 0.01 and *p* < 0.001 levels, respectively. BP, boll plant^−1^; Corr, correlation; LW, lint weight; MDA, malondialdehyde; PH, plant height; RDW, root dry weight; RWC, relative water content; and SDW, shoot dry weight.

Correlation analysis was performed to find out the positive and negative relationships among different morphological, physiological, and yield attributes of four investigated cotton cultivars. The upper triangle of the correlation matrix revealed that plant height, shoot dry weight, bolls per plant, lint weight, seed weight, and seed cotton weight had (*p* < 0.05, *p* < 0.01 or *p* < 0.001) positive correlations with each other (Figure 5). However, root length and root dry weight had no significant relationship with other traits. Specifically, lint weight displayed a positive correlation (*p* < 0.05 and *p* < 0.01) with seed cotton weight and seed weight in the case of all the cotton cultivars, except CB-12 for seed weight. Furthermore, the diagonal part is presented to visualize the density plots of the investigated traits. The X axis of each density plot indicates the values of the variables, whereas the Y axis shows the relative probabilities of an area under the curve. The highest density of the values of the traits is shown in the area under the curve around the peak of the density plots. The density plots revealed that all the studied traits were influenced by the different cotton cultivars as represented by the densities as well as their magnitude. Among the investigated cotton cultivars, Rupali-1 (purple color) and CB-12 (red color) showed the highest peaks in most of the studied traits (Figure 5).

### 2.5. Principal Component (PC) Analysis (PCA) among the Variables of Four Cotton Cultivars under Different WL Durations

**Figure 6:**
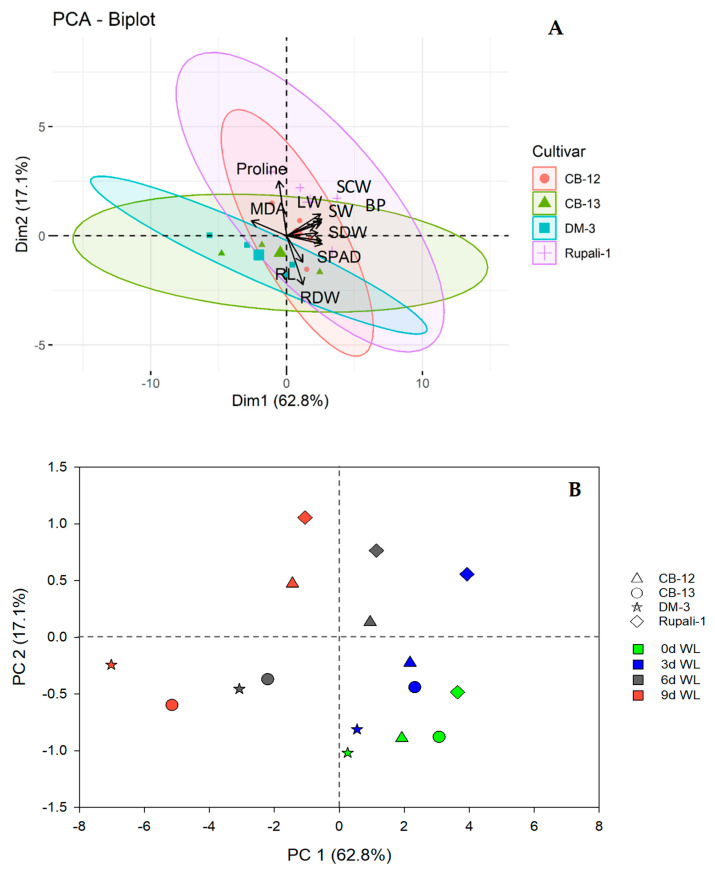
Principal component (PC) analysis (PCA) shows PC1 and PC2. (**A**) The bi-plot represents the relationship among the investigated traits of the four elite cotton cultivars. (**B**) The cluster shows the tolerance levels of the cotton cultivars under various WL conditions. BP, boll plant^−1^; LW, lint weight; PH, plant height; RDW, root dry weight; RL, root length; SCW, seed cotton weight; SDW, shoot dry weight; SW, seed weight; and WL, waterlogging.

The PCA results of different variables are presented in Figure 6. The first and second PCs explained about 80% of the total variations of the variables estimating 62.8 and 17.1%, respectively. All variables had positive loading on the first component except MDA content and root dry weight, whereas a number of variables like lint weight, seed weight, seed cotton weight, SPAD, RWC, boll plant^−1^, plant height, and shoot dry weight had positive loading on the second component, which indicated the highly contributing traits among the variables. As a result, the association between variables was created. Specifically, lint weight, seed weight, and shoot dry weight were closely associated with plant height, boll plant^−1^, seed cotton weight, SPAD, RWC, and proline content. In considering genotypic effect, Rupali-1 and CB-12 exhibited better yield performance and leaf proline accumulation and more negative relation with MDA content than CB-13 and DM-3 (Figure 6A). Moreover, the four cotton cultivars showed the highest tolerance level at the top right section of the scatter plot and moderate tolerance and sensitivity at the top left and lower right section, respectively, of the scatter plot against different WL conditions. Contrastingly, the lower left section indicates the most sensitive cotton cultivars against different WL levels (Figure 6B). These results together indicated that Rupali-1 and CB-12 showed higher levels of tolerance than CB-13 and DM-3 at 6 d and 9 d of WL (Figure 6B).

## 3. Discussion

WL has become more frequent and unpredictable worldwide during the last few decades [40] and is considered a major constraint affecting crop production in many countries in the world [28,41]. Cotton plants are particularly sensitive to WL at reproductive phases [23]. The current study investigated the effect of WL on several growth, physiological, and yield-related parameters of four elite cotton cultivars under field conditions. Our observation revealed that the exposure of cotton plants to 6 d and 9 d of WL significantly reduced the growth attributes of cotton, including plant height, number of sympodial branches, and shoot dry weight (Figure 1C,E and Figure 2A). Among the four cotton cultivars, Rupali-1 and CB-12 exhibited the lowest reductions in plant height, number of sympodial branches, and shoot dry weight. As long as the WL progressed, the root dry weight in Rupali-1 continued to decrease, showing the highest decrease by 65% at 9 d of WL (Figure 2B). On the other hand, CB-12 and CB-13 showed statistically similar root dry weight even at 9 d of WL compared with their respective 0-d control. The lowest root and the highest shoot dry matter accumulations of Rupali-1 at 9 d of WL might be linked to the fact that Rupali-1 devoted more energy for dry matter partitioning toward the reproductive sink rather than using energy for root development under WL conditions at the reproductive stage. A wealth of studies also reported that WL significantly affected the growth and development of cotton plants, including plant height, root length, and shoot and root biomass [6,15,22,23,34,42]. It has also been reported that the deleterious effect of WL led to late maturity, boll abscission, lower yield, and reduced fiber quality [21,23,29,37,38,39,43,44,45,46]. In fact, WL conditions create an imbalance between the production and consumption in plants’ carbohydrates. Eventually, the lack of energy supply caused by anaerobic respiration under hypoxia hinders plant growth and development, which may lead to plant death, especially when it is coupled with an accumulation of toxic metabolites [1,12,21]. In the present study, all the four investigated cotton cultivars showed significant reductions in yield and yield-contributing parameters, particularly after being exposed to 6 d and 9 d of WL, which was presumably linked to low energy supply under WL conditions [39]. However, no significant differences were observed at 3 d of WL compared with the non-waterlogged plants (Figure 1 and Figure 2).

Likewise, in the case of other yield-contributing parameters, the highest numbers of boll and individual boll weight were observed in CB-12 and Rupali-1 at 9 d of WL. Furthermore, the highest seed cotton weights, lint weights, and seed weights were recorded in CB-12 and Rupali-1 at 6 d and 9 d of WL, whereas no significant variation was found at 3 d of WL when compared with their respective controls. Furthermore, CB-13 and DM-3 exhibited the highest yield reductions, whereas CB-12 and Rupali-1 showed the highest stability in yield at 6 d and 9 d of WL. As a whole, our data revealed that CB-12 and Rupali-1 have the highest potential to adapt to all the tested WL conditions (Figure 4A,C–E). A previous study also reported that boll number was significantly reduced after being exposed to WL [46]. In another study, cotton yield was found to be reduced by 27–30% at 4 d to 9 d of WL exposure [47]. Jiang et al. [48] reported that 10 d of WL significantly affected the number of bolls in cotton plants, leading to a 42% reduction in fiber yield.

Here, we investigated some important physiological and biochemical parameters to correlate the WL-affected growth and yield-contributing data obtained from four cotton cultivars (Figure 3). We observed that RWC and chlorophyll intensity values (according to SPAD reading values) were significantly reduced, particularly in CB-12 and DM-3, at 6 d and 9 d of WL (Figure 3C,D). In contrast, Rupali-1 and CB-12 exhibited the lowest reduction percentages in RWC and SPAD values at the same WL exposure (i.e., 6 d to 9 d) compared with their 0-d control and 3 d of WL (Figure 3C,D). It is well known that an increase in MDA content is considered an indicator of cellular oxidative damage that is caused by several environmental stress conditions [49]. Plants can defend themselves against various environmental stresses, including WL, by upregulating cellular proline levels and lowering the MDA production to maintain cell membrane stability, detoxifying reactive oxygen species (ROS) [50], and maintaining osmotic pressure [51,52,53]. In the present study, the proline and MDA levels were significantly raised with the increasing WL duration (Figure 3A,B). The highest leaf proline contents were recorded in Rupali-1 and CB-12 at 9 d of WL compared with their 0-d control, suggesting that the higher accumulation of leaf proline might contribute to the synthesis of cell wall-bound proline-rich proteins, which would eventually contribute to maintaining membrane integrity [54] and RWC in these two cotton cultivars. Furthermore, the lower increase of MDA contents in Rupali-1 and CB-12, compared with CB-13 and DM-3 at 9 d of WL (Figure 3B), indicated lower cellular oxidative damage in Rupali-1 and CB-12 under WL stress, i.e., their better adaptation to WL. Previous studies also reported that WL significantly enhanced MDA and proline contents in plants [46,55]. Several studies also reported that WL-affected wheat (*Triticum aestivum*) plants had heightened leaf proline contents, which facilitated the maintenance of proper RWC to sustain under WL [56,57,58,59]. Furthermore, from the correlation analysis and PCA, Rupali-1 and CB-12 were shown to sustain under the highest levels of WL and secured the highest yields. Thus, the present findings suggest that Rupali-1 and CB-12 would be the ideal cotton cultivars for WL-prone areas.

## 4. Materials and Methods

### 4.1. Treatments, Plant Materials, and Culture Conditions

The experiment was carried out at the Patuakhali Science and Technology University (22°27’51.8” N 90°23’14.9” E). The experiment included two factors: factor (a) with four elite cotton cultivars, i.e., CB-12, CB-13, Rupalli-1, and DM-3; and factor (b) with four WL durations, i.e., 0 d (normal irrigation as control), 3 d, 6 d, and 9 d of WL. The cotton cultivars were collected from the Cotton Development Board (CDB), Bangladesh. Cotton seeds were germinated separately in soil having all the required nutrient elements. The seedlings at the trifoliate stage (approximately 30-day-old seedlings) with uniform growth were transplanted into the pots at one plant per pot. The experiment was set up in concrete pots using the soil culture method, and the soil was sandy loam. The pots were cylindrical, made of cement, and 40 × 36 × 55 cm. Pots received a basal application of fertilizer at 0.77 g urea, 6.95 g triple super phosphate, 1.55 g gypsum, 0.30 g MgSO_4_, and 1 kg cow dung, as per the recommendation of the Cotton Development Board [19]. For creating WL conditions, the holes at the bottom of the pots were sealed off with cement. Water was added to the pots through a plastic pipe using an electric pump. The water depth in all the pots was kept at 5 cm above the soil surface during the experiment [23]. The WL condition (5 cm water above soil surface) exposed to the cotton plants at their reproductive stage (during flowering and boll formation) was created through irrigation and maintained for the expected duration as mentioned above in factor b. After the designated period of WL, excess water was drained out to ensure normal growth conditions.

### 4.2. SPAD Readings

SPAD reading values were determined immediately after the WL treatment was completed at boll formation stage using a SPAD meter (SPAD-502 Plus, Konica Minolta, Japan). The representative SPAD reading values were obtained from the mean value of the three top most fully expanded leaves.

### 4.3. Leaf RWC

For measuring the leaf RWC, the fully expanded cotton leaf sample was collected from the topmost position of the plant after completing the WL treatment (3 d, 6 d or 9 d) at the boll formation stage. Briefly, the fresh weights of the collected leaves were taken using weighing balance. Then, each of the collected leaf samples were immersed into water for 24 h to obtain the turgid weight. Subsequently, each leaf sample was placed in a paper envelope and oven-dried at 72 °C for 72 h, and leaf dry weight was then recorded and %RWC was determined using the following formula [60]:

(1) $$\mathrm{RWC}\;\left(\%\right)=\left(\frac{\mathrm{FW}-\mathrm{DW}}{\mathrm{TW}-\mathrm{DW}}\right)\times 100$$

where, FW = fresh weight, TW = turgid weight, DW = dry weight.

### 4.4. Leaf Proline Content

The free proline content was determined according to Bates et al. [61]. Briefly, 0.5 g frozen leaf sample was homogenized in 10 mL of 3% sulphosalicylic acid at 4 °C. The extract was centrifuged for 10 min in a centrifuge machine at 3000 rpm. In a test tube, 2 mL filtrate, 2 mL acid-ninhydrin, and 2 mL glacial acetic acid were added and left at 100 °C for 1 h. The reaction was terminated on ice. The reaction mixture was extracted with 4 mL toluene. The chromophore containing toluene was separated from the hydrated phase. The absorbance at 520 nm was determined spectrophotometrically (T60 UV-Visible Spectrophotometer, Beijing Karaltay Co., Ltd., Beijing, China). Proline concentrations were calculated according to the standard curve and expressed as µmol g^−1^ leaf fresh weight (FW).

### 4.5. MDA Quantification

MDA is an indication of cellular lipid peroxidation. It was measured using the method of Heath and Packer [62] and Ali et al. [63]. In brief, 0.2 g fresh leaf sample and 1.5 mL of 0.1% trichloro-acetic acid (TCA) were homogenized together. After that, the solution was centrifuged for 15 min at 11,500× *g* at 4 °C, and the supernatant was moved into another tube. Then, 0.4 mL supernatant was added to 1 mL of the reaction mixture (RM). Following this, 1 mL of RM was combined with 0.4 mL of 0.1% TCA for blank. In a bath of boiling water, the tube was incubated at 95 °C for 30 min. The tube was then placed in an icebox to stop the reaction and centrifuged again for 30 min at 10,000× *g*. Finally, the absorbance of the collected supernatant was measured at 532 and 600 nm using a spectrophotometer.

Calculation:

(2) $$\mathrm{MDA}\;\mathrm{content}\;\left({µ\mathrm{mol}\;g}^{-1}\;\mathrm{FW}\right)=\frac{\left(D532-D600\right)\times \mathrm{volume}\;\mathrm{of}\;\mathrm{total}\;\mathrm{mixture}\;\left(0.4\;\mathrm{mL}\;\mathrm{supernatant}+1\;\mathrm{mL}\;\mathrm{of}\;\mathrm{RM}\right)\times 1000}{\mathrm{Extinction}\;\mathrm{co}-\mathrm{efficient}\;\left(155{\;\mathrm{mM}}^{-M}{\mathrm{cm}}^{-1}\right)\times \mathrm{sample}\;\mathrm{weight}\;\left(0.2\;g\right)}$$

The extinction co-efficient of mM is converted to µM by multiplying with 1000 where D532 and D600 are absorbance readings at 532 and 600 nm, respectively.

### 4.6. GOT

The GOT percentage per cotton plant was estimated using the following formula:

(3) $$\mathrm{GOT}\;\left(\%\right)=\left(\frac{\mathrm{weight}\;\mathrm{of}\;\mathrm{lint}}{\mathrm{weight}\;\mathrm{of}\;\mathrm{seed}\;\mathrm{cotton}}\right)\times 100$$

### 4.7. Growth and Yield Attributes

Plant height and root length were determined at the end of the experiment using a measuring tape. Shoots and roots were collected after harvest and oven-dried at 70 °C for 72 h to determine dry weight. Seed cotton, lint, and seed weights were measured using an electrical balance.

### 4.8. Statistical Analysis and Interpretation of Data

To test the WL effect at varying durations (0 d, 3 d, 6 d, and 9 d) on the reproductive stage of four cotton cultivars (CB-12, CB-13, Rupalli-1, and DM-3), data of various growth, physiological, and biochemical parameters were subjected to analysis of variance (ANOVA) and means were compared using Tukey’s honestly significant difference (HSD) post hoc test using the computer-based statistical program JMP 14 (SAS Institute Inc., Cary, NC, 1989–2019) following the basic principles. We used Sigmaplot v14 from Systat Software, Inc., San Jose, CA, USA (www.systatsoftware.com accessed on 28 December 2022) to present the data, and program R (R for Windows, v4.1.2) was used for PCA and Spearman correlation analysis.

## 5. Conclusions

The present study demonstrated that the selected elite upland cotton cultivars were variably affected by different levels of WL. Notably, the Rupali-1 and CB-12 showed the best performance with respect to overall growth and yield parameters studied under WL conditions. Higher WL adaptation of the Rupali-1 and CB-12 were found to be linked to higher RWC, SPAD reading values (reflecting chlorophyll content) and leaf proline content, and lower cellular MDA content even at 6 d and 9 d of WL exposure. On the other hand, CB-13 and DM-3 were identified as sensitive cultivars when exposed to 6 d and 9 d of WL, as evidenced by their poor morpho-physiological and yield attributes. In addition, the Spearman correlation analysis and PCA revealed that CB-12 and Rupali-1 had the highest coefficient values in yield and physiological attributes at 9 d WL, whereas CB-13 and DM-3 appeared to be sensitive cultivars in response to the same WL durations. Thus, CB-12 and Rupali-1 might be suitable for cultivation in low-lying WL-prone areas.

## Data Availability

Not applicable.
